# Ferumoxytol-enhanced magnetic resonance imaging assessing inflammation after myocardial infarction

**DOI:** 10.1136/heartjnl-2016-311018

**Published:** 2017-06-22

**Authors:** Colin G Stirrat, Shirjel R Alam, Thomas J MacGillivray, Calum D Gray, Marc R Dweck, Jennifer Raftis, William SA Jenkins, William A Wallace, Renzo Pessotto, Kelvin HH Lim, Saeed Mirsadraee, Peter A Henriksen, Scott IK Semple, David E Newby

**Affiliations:** 1 British Heart Foundation/University Centre for Cardiovascular Science, University of Edinburgh, Edinburgh, UK; 2 Clinical Research Imaging Centre, University of Edinburgh, Edinburgh, UK; 3 Clinical Research Facility, University of Edinburgh, Edinburgh, USA; 4 Department of Pathology, University of Edinburgh, Edinburgh, UK; 5 Department of Cardiothoracic Surgery, Royal Infirmary of Edinburgh, Edinburgh, UK

**Keywords:** Cardiac, MRI, Myocardial Infarction, Molecular Imaging, Inflammation, USPIO

## Abstract

**Objectives:**

Macrophages play a central role in the cellular inflammatory response to myocardial infarction (MI) and predict subsequent clinical outcomes. We aimed to assess temporal changes in cellular inflammation and tissue oedema in patients with acute MI using ultrasmallsuperparamagnetic particles of iron oxide (USPIO)-enhanced MRI.

**Methods:**

Thirty-one patients were recruited following acute MI and followed up for 3 months with repeated T2 and USPIO-enhanced T2*-mapping MRI. Regions of interest were categorised into infarct, peri-infarct and remote myocardial zones, and compared with control tissues.

**Results:**

Following a single dose, USPIO enhancement was detected in the myocardium until 24 hours (p<0.0001). Histology confirmed colocalisation of iron and macrophages within the infarcted, but not the non-infarcted, myocardium. Following repeated doses, USPIO uptake in the infarct zone peaked at days 2–3, and greater USPIO uptake was detected in the infarct zone compared with remote myocardium until days 10–16 (p<0.05). In contrast, T2-defined myocardial oedema peaked at days 3–9 and remained increased in the infarct zone throughout the 3-month follow-up period (p<0.01).

**Conclusion:**

Myocardial macrophage activity can be detected using USPIO-enhanced MRI in the first 2 weeks following acute MI. This observed pattern of cellular inflammation is distinct, and provides complementary information to the more prolonged myocardial oedema detectable using T2 mapping. This imaging technique holds promise as a non-invasive method of assessing and monitoring myocardial cellular inflammation with potential application to diagnosis, risk stratification and assessment of novel anti-inflammatory therapeutic interventions.

**Trial registration number:**

Trial registration number: 14663. Registered on UK Clinical Research Network (http://public.ukcrn.org.uk) and also ClinicalTrials.gov (https://clinicaltrials.gov/ct2/show/NCT02319278?term=DECIFER&rank=2).

## Introduction

More than one person every minute suffers a myocardial infarction (MI) in the USA.^1^ Despite improved interventional and medical treatments, mortality rates following MI remain high and many develop heart failure.^1^ Postinfarct inflammation plays a key role in the recovery of cardiac function,^2 3^ and it is a target for therapeutic manipulation to improve clinical outcomes.

After early neutrophil infiltration, monocyte-derived macrophages dominate the cellular infiltrate in the first 2 weeks following MI and sequentially coordinate digestion of damaged tissue and promotion of infarct healing.^2 3^ Optimal post-MI recovery is determined by the balance between proinflammatory and reparative macrophages, with uncontrolled early inflammation leading to adverse functional recovery.^2–5^

Ultrasmall superparamagnetic particles of iron oxide (USPIO) consist of an iron oxide core surrounded by a carbohydrate or polymer coating and are small enough to extravasate through diseased microvessels, where they are engulfed and concentrated by tissue-resident macrophages.^6^ Accumulation of USPIOs reduces T2* decay time and creates signal deficits that can be quantified and visualised using T2* MRI.^7 8^ Thus, USPIO-enhanced MRI can detect tissue-resident macrophage activity and identify cellular inflammation within tissues.

In current practice, T2-weighted MRI is used to evaluate myocardial oedema after MI.^9 10^ However, these imaging techniques assess myocardial free water content and not active cellular inflammation. Development of a reliable non-invasive imaging technique capable of directly detecting myocardial cellular inflammation would be a major advance and could potentially facilitate risk stratification and therapeutic targeting of macrophages immediately after MI. Furthermore, this technique could provide diagnostic information, serial disease monitoring and a measure of treatment response in other conditions mediated by inflammatory cell infiltration of the heart.

In a recent proof-of-principle pilot study,^7^ we successfully detected inflammation following acute MI using USPIO-enhanced MRI. USPIO uptake, defined as the increase in R2* (1/T2*) 24 hours following administration, was seen in the infarct zone within the heart. In this study, we hypothesised that we could track the time course of cellular inflammation in the 3-month period following acute MI using USPIO-enhanced MRI. Our primary aims were, first, to determine the duration and distribution of USPIO enhancement in tissues following a single infusion and, second, to assess the duration of macrophage infiltration of the heart by tracking myocardial USPIO enhancement using repeated USPIO administration, and compare this to measures of myocardial oedema using T2 mapping.

## Methods

This was an open-label observational cohort study. Patients were recruited within 7 days of acute MI. The study was performed in accordance with the Declaration of Helsinki, the approval of the local research ethics committee and the written informed consent of all participants.

### Subjects

Participants were aged 18–80 years and had sustained a recent MI according to the third universal definition of MI,^11^ with 12-hour plasma troponin I concentration ≥5000 ng/L. Exclusion criteria were known critical stenosis (>95%) of left main stem, ongoing symptoms of angina, heart failure (Killip class ≥2), renal failure (estimated glomerular filtration rate <30 mL/min/1.73 m^2^) and contraindication to MRI or ferumoxytol infusion.

### Ultrasmall superparamagnetic particles of iron oxide

Intravenous infusion of USPIO (ferumoxytol, Fe core 3.25 nm, total particle diameter 17–31 nm, 4 mg/kg; Rienso, Takeda Italia, Italy) was performed immediately following the baseline magnetic resonance scan over at least 15 min using a concentration of 2–8 mg/mL, diluted in 0.9% saline or 5% dextrose. Haemodynamic monitoring was conducted throughout.

### Study protocol

MRI and tissue histology were performed as described in the online supplementary material. A variable study protocol was used that was purposely designed for imaging at different time points after acute MI. Over a 3-month period following MI, patients received up to seven MRI scans and up to three infusions of USPIO. Two principal analyses were performed using data from the same participants.

**Figure 1:**
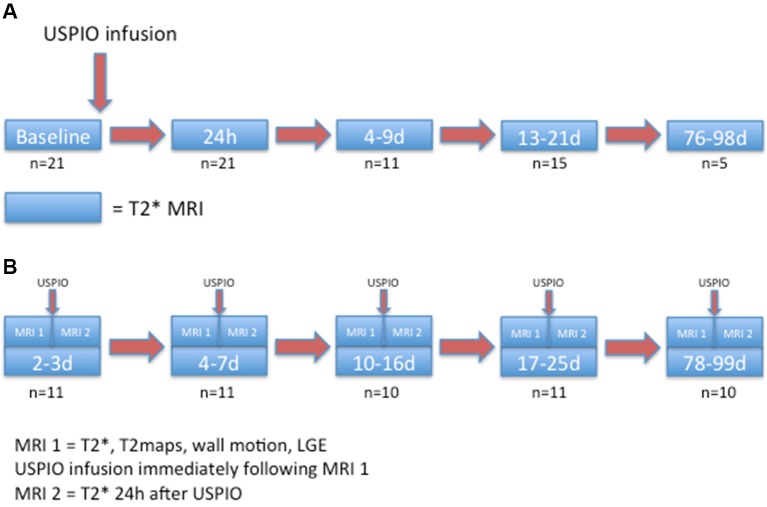
(A) Duration and distribution of USPIO enhancement after single-dose administration in the first week after MI. (B) Repeated myocardial uptake of USPIO following MI. MI, myocardial infarction; USPIO, ultrasmall superparamagnetic particles of iron oxide.

First, to assess the duration of a single dose of USPIO, R2* values of only the participants who first received USPIO within 7 days of MI (21/30) were included in this analysis. They were followed on all subsequent MRI scans prior to receiving any further doses of USPIO (figure 1A). On receiving further USPIO, data were censored from the point of the second USPIO administration, hence the reduction in numbers during the course of the study.

Second, to assess the duration of myocardial macrophage activity, USPIO uptake was compared using repeated USPIO infusions over the 3-month follow-up period. All patients were included in this analysis. USPIO uptake was calculated by subtracting the baseline R2* value from values obtained at 24 hours following USPIO infusion (figure 1B). This allowed repeated assessment of myocardial USPIO uptake.

Following clinical guidance for the administration of ferumoxytol, participants were allowed a maximum of two doses of USPIO in a 1-month period, and a total of three over the 3-month follow-up period to prevent iatrogenic iron overload. Therefore, USPIO-enhanced data were collected up to three times for each patient, and it was not possible to perform repeated USPIO-enhanced imaging at every time point in the same patient.

Finally, to assess myocardial oedema, T2 values were compared on unenhanced scans (prior to and at least 7 days following USPIO administration). All patients were included in this analysis.

### Image analysis

All T2*-weighted multigradient-echo images for each patient were analysed using Circle CVI software (Circle CVI42, Canada). An experimentally determined threshold used in previous work^7^ for the coefficient of determination (r^2^>0.85) was used to exclude data that did not have an acceptable exponential decay when signal intensity was plotted against echo time. The relaxation rate, R2*, is the inverse of the mean T2*, and was calculated to assess the uptake of USPIO for each region of interest (ROI), where the higher the value, the greater the USPIO accumulation.

Late gadolinium enhancement (LGE), ventricular volume and functional analyses were determined manually using QMass software (Medis Medical Imaging Systems, Leiden, The Netherlands).

### ROI selection

LGE images at the 3-month time point were used to determine the distribution and anatomy of the myocardial infarct, and served as reference images to which each series of T2 and T2* scans at each separate visit could be visually coaligned. This allowed myocardial ROIs to be selected on the reference short-axis LGE images and manually applied to T2* images corresponding to (1) infarct zone (defined by the short axis slice showing greatest LGE volume), (2) peri-infarct zone (half segment width immediately adjacent to LGE), and (3) remote myocardium (at least half segment width, opposite the infarct zone on the same short axis slice, and at least one full segment clear of any LGE). Other selected regions included (4) liver, (5) spleen, (6) bone marrow, (7) kidney, (8) blood pool, (9) aortic wall (inside arch) and (10) skeletal muscle.

T2 values were measured immediately prior to USPIO administration. USPIO-enhanced data were collected 24 hours following USPIO administration.

### Statistical analysis

All statistical analyses were performed with GraphPad Prism, V.6 (GraphPad Software, San Diego, California, USA). To assess the duration of USPIO enhancement in tissues following single administration in the first week after MI, R2* values on subsequent MRI scans (prior to any further USPIO dosing) were compared using one-way analysis of variance (ANOVA) with Tukey’s post hoc test for multiple comparisons.

To assess variation and duration of myocardial inflammation, USPIO uptake (R2* increase from pre to 24 hours following USPIO) was compared using one-way ANOVA with Tukey’s post hoc test for multiple comparisons (repeated measures where appropriate). As a further method to assess USPIO accumulation within the heart, post-USPIO R2* 24 hours after administration (without baseline R2* subtraction) was compared in the same way.

Finally, to assess variation and duration of myocardial oedema, T2 values for infarct, peri-infarct and remote myocardium were compared again using one-way ANOVA with Tukey’s post hoc test for multiple comparisons (repeated measures where appropriate). Statistical significance was defined as two sided, p<0.05.

## Results

Thirty-one patients were recruited although 1 patient was excluded due to claustrophobia. A total of 147 MRI scans and 54 infusions of ferumoxytol were completed during the course of the study. Administration of ferumoxytol was well tolerated with no adverse reactions reported during or immediately after administration.

Participants were predominantly middle-aged men with current or previously diagnosed hypercholesterolaemia or smoking habit (table 1). Around half of the participants were hypertensive and had a family history of premature coronary heart disease. Twenty-five patients had ST-segment elevation MI, 5 patients had non-ST-segment elevation MI, 26 underwent percutaneous coronary intervention, and 4 had coronary artery bypass grafting (CABG) surgery. The mean elevation in plasma troponin was 31 200 ng/L.

**Table 1:** Participant characteristics

Number	30
Age (years)	58.4±9.8
Sex	Male—29 (97) Female—1 (3)
Weight (kg)	88.7±14.8
Risk factors	
Hypertension	13 (43)
Diabetes mellitus	8 (27)
Hypercholesterolaemia	27 (90)
Family history	14 (47)
Current or ex-smoker	26 (87)
Infarct characteristics	
STEMI	25 (83)
Anterior	10 (33)
Inferior	15 (50)
Lateral	0 (0)
Diagnostic ECG to reperfusion (STEMI, min)	72.5±16.8
NSTEMI	5 (17)
PCI	26 (87)
Baseline ejection fraction (%)	48.5±11.2
Infarct size (% of LV mass)	31.6±19.5
Infarct volume (mL)	43.9±27.5
Plasma troponin (ng/L)	31 229±18 563
Subsequent CABG/biopsy)	4/3

Mean±SD, N (%).

CABG, coronary artery bypass grafting; LV, left ventricle; NSTEMI, non-ST-elevation myocardial infarction; PCI, percutaneous coronary intervention; STEMI, ST-elevation myocardial infarction.

### Enhancement after single USPIO dose

Twenty-one of the 30 participants received USPIO in the first week after MI and were included in this analysis.

**Figure 2:**
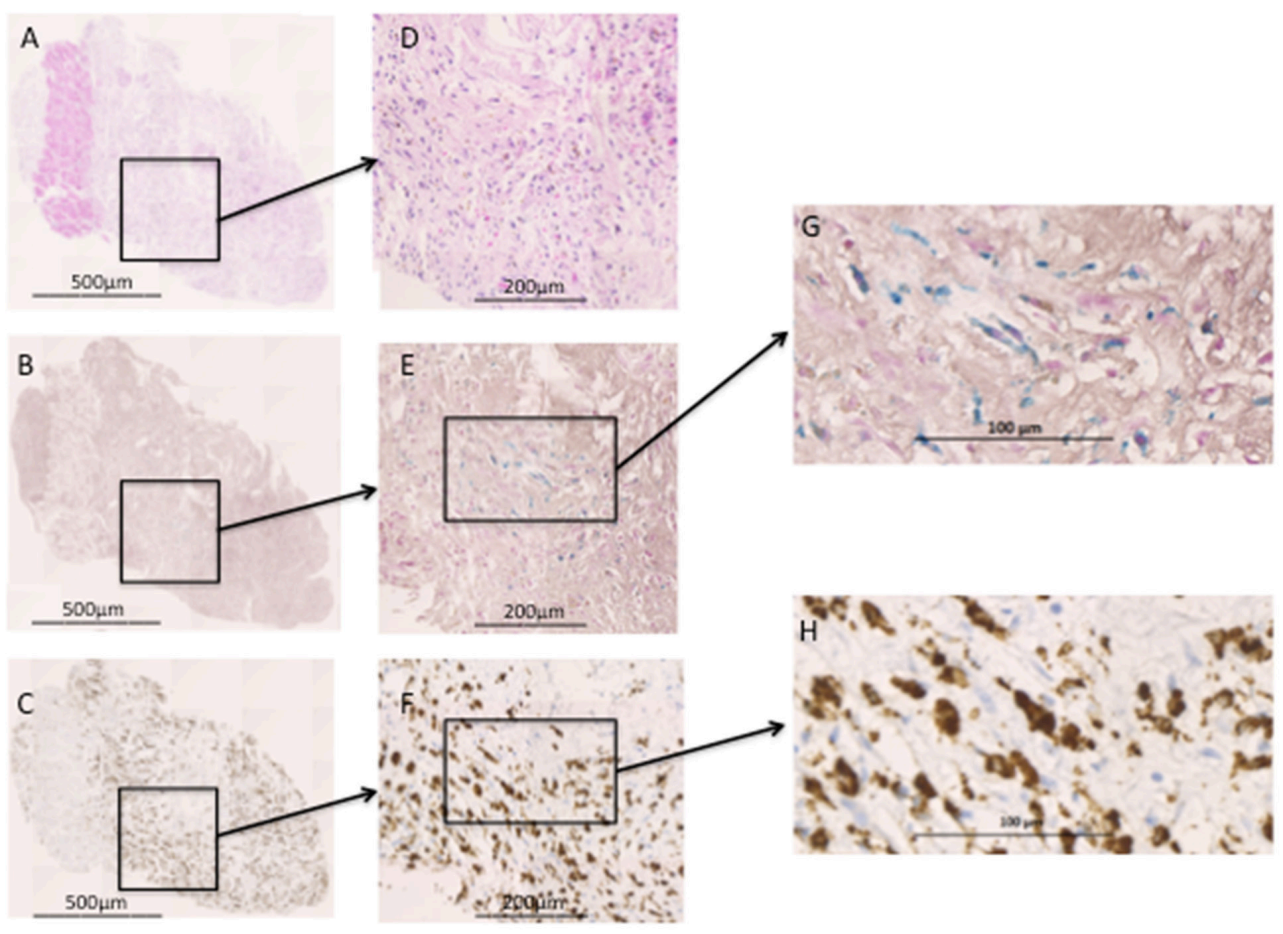
Histology section of trucut biopsy from infarcted myocardium. (A) Haematoxylin and eosin (×5 magnification) stain displaying a thin region of viable healthy myocardium (strip of dark pink) surrounded by infarcted myocardium (lighter pink). (D) Infarcted myocardium shows an abundance of inflammatory cells and early granulation formation. (B) Prussian blue (×5) staining revealing intracellular iron (E, ×20 and G, ×50), not seen in the region of healthy myocardium. (C) CD68 (×5) staining revealing macrophages within the infarcted myocardium (F, ×20 and H, ×50), again not seen in healthy myocardium. G+H show colocalisation of iron within macrophages.

USPIO was detected by an increase in R2* in all myocardial regions, blood pool, kidney and aortic wall until 24 hours, being cleared by 4–9 days (figure 2, online supplementary figure 1 and online supplementary table 1). USPIO was detected in bone marrow until 4–9 days and in liver and spleen until 13–21 days. There was no change in R2* following USPIO infusion in skeletal muscle.

10.1136/heartjnl-2016-311018.supp1 Supplementary data

Myocardial biopsies were taken from three of four patients undergoing CABG and revealed an abundance of inflammatory cells and early granulation tissue in keeping with a region of healing myocardium from around the infarct zone. Histological staining revealed colocalisation of the presence of iron (Prussian blue) and macrophages (CD68). No staining for iron or macrophages was seen within the adjacent regions of healthy myocardium (figure 2).

### Time course of enhancement with repeated USPIO administration

**Figure 3:**
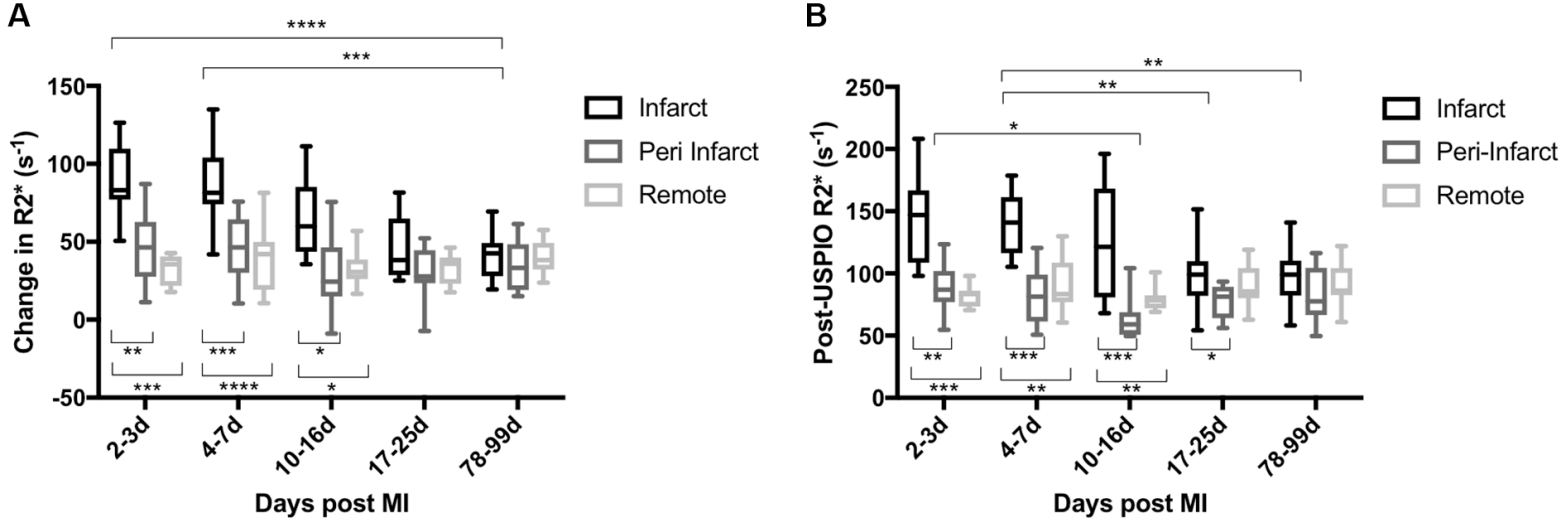
(A) USPIO uptake in myocardium after MI with repeated USPIO administration. (B) R2* 24 hours following USPIO (without subtraction of baseline values). Time course variation in USPIO uptake was seen in the infarct zone peaking at days 2 and 3. No variation of USPIO uptake was seen in peri-infarct and remote myocardium. Compared with remote myocardium, increased USPIO uptake was seen in the infarct zone until days 10–16 post MI. (****p<0.0001, ***p<0.001, **p<0.01, *p<0.05). Compared with remote myocardium, greater post-USPIO R2* was seen in the infarct zone until days 10–16 post MI. (***p<0.001, **p<0.01, *p<0.05). A+B: n = 11 (days 2–3, 4–7 and 17–25), 10 (days 10–16) and 10 (days 78–99). MI, myocardial infarction; USPIO, ultrasmall superparamagnetic particles of iron oxide.

**Figure 4:**
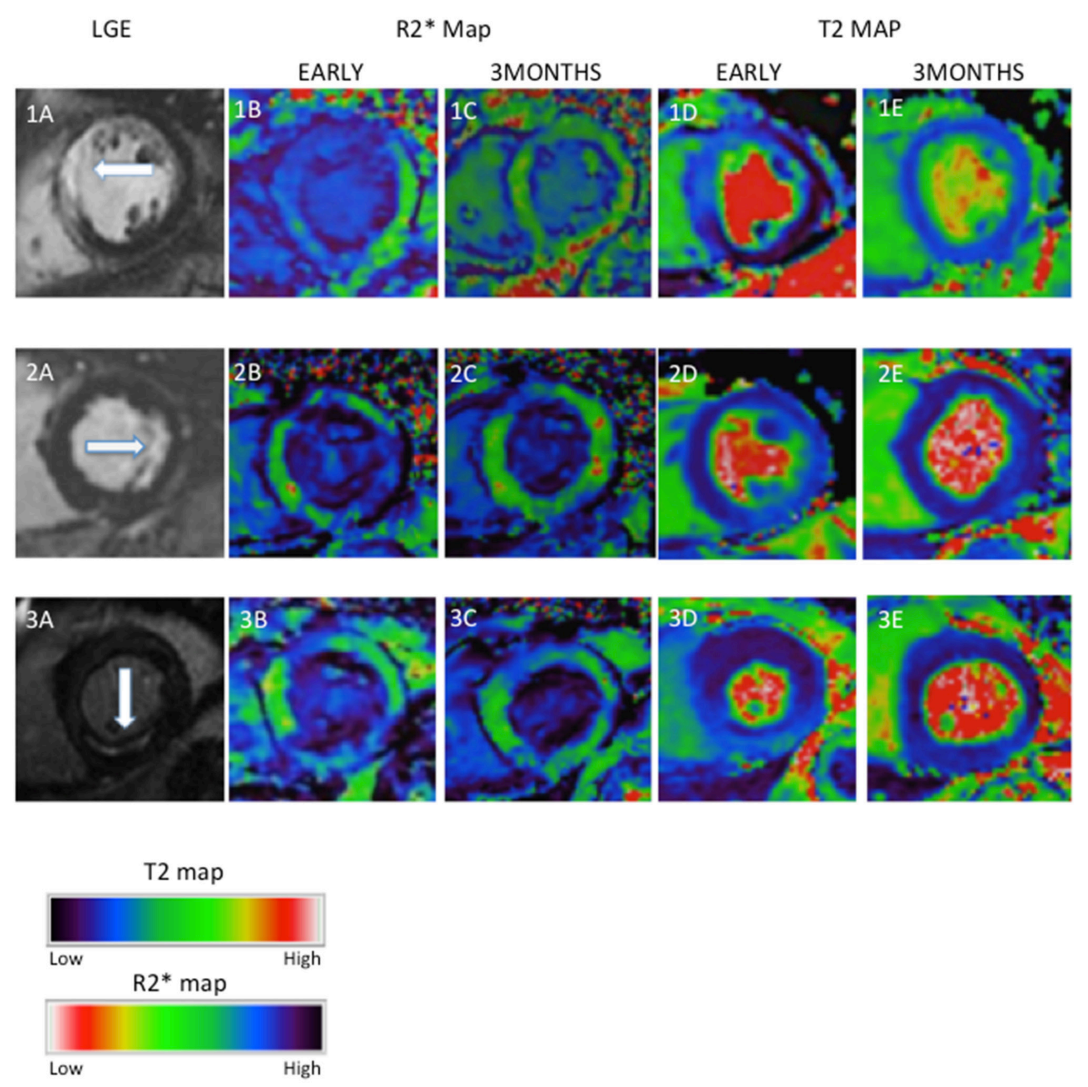
Examples of myocardial oedema and USPIO enhancement in the infarct zone after MI. Three examples of MI (1—anteroseptal, 2—lateral and 3—inferior) illustrating LGE, USPIO enhancement (R2* map) and oedema (T2 map) at early (up to 10 days) and late (3 months) time points. Early inflammation and oedema seen on R2* (dark region) and T2 maps (light region), respectively, have improved or resolved by 3 months. LGE, late gadolinium enhancement; MI, myocardial infarction; USPIO, ultrasmall superparamagnetic particles of iron oxide.

There were no differences in baseline (pre-USPIO) R2* value between infarct and remote myocardium at all time points. Time course variation in USPIO uptake was seen in the infarct zone over the 3-month period, peaking at days 2 and 3 (figures 3a, 4 and online supplementary table 2a). There was no time course variation in USPIO uptake in the peri-infarct and remote myocardium or extra-cardiac tissues over the 3-month follow-up period.

Compared with remote myocardium, greater USPIO uptake was seen in the infarct zone until days 10–16 (mean +56 s^−1^; 95% CI 33 to 79; p<0.001, +47 s^−1^; 95% CI 31 to 62; p<0.0001, and +33 s^−1^; 95% CI 4 to 61; p<0.05 at 2–3 days, 4–7 days and 10–16 days, respectively) but not thereafter (figure 3a). Similarly, comparing R2* 24 hours following USPIO (without baseline R2* subtraction), greater USPIO uptake was again evident in infarct myocardium compared with remote myocardium until 10–16 days (mean +60 s^−1^; 95% CI 33 to 86; p<0.01, +49 s^−1^; 95% CI 21 to 77; p<0.01, and +45 s^−1^; 95% CI 2 to 87; p<0.05 at 2–3 days, 4–7 days and 10–16 days, respectively, figure 3b and online supplementary table 2b).

### Assessment of myocardial oedema using T2 mapping

**Figure 5:**
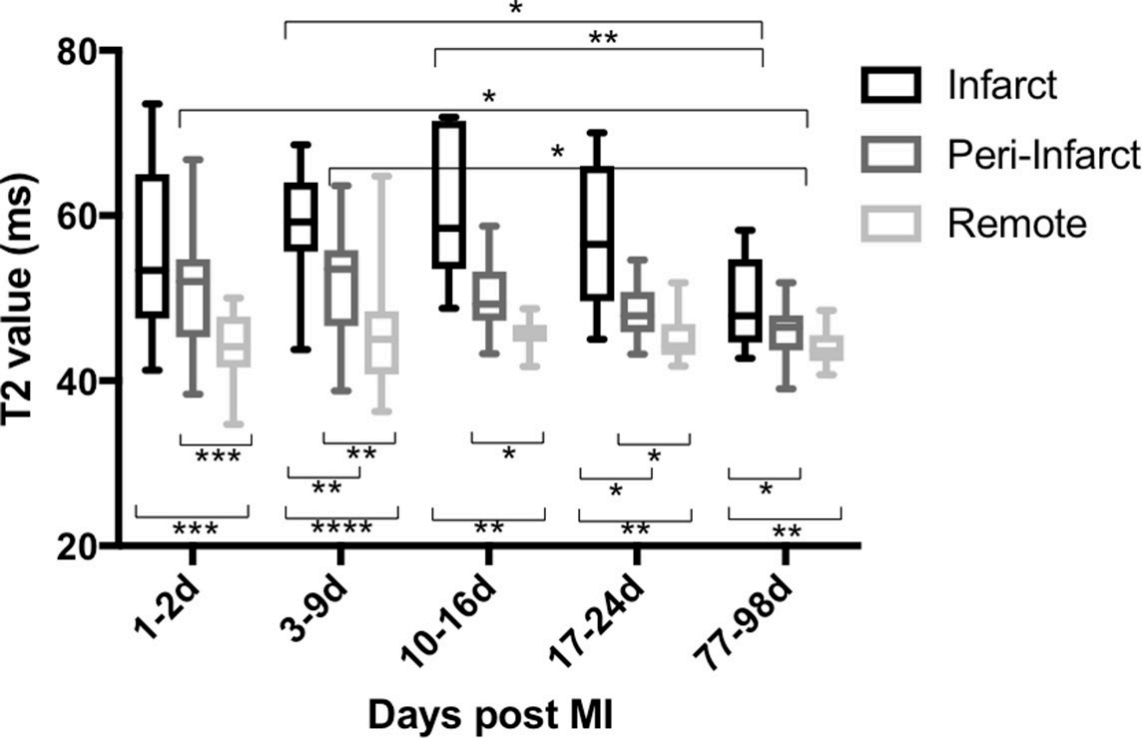
T2 value in the myocardium after MI. Time course variation in infarct and peri-infarct T2 was seen in the 3-month period following MI, both peaking at days 3–9. Compared with remote myocardium, higher T2 was seen in the infarct zone throughout the duration of the study and in the peri-infarct zone until days 17–24 (****p<0.0001, ***p<0.001, **p<0.01, *p<0.05); n = 15 (days 1–2), 14 (days 3–9 and 77–98), 8 (days 10–16) and 10 (days 17–24). MI, myocardial infarction.

Following acute MI, time course variation in infarct and peri-infarct T2 values was seen (see figure 5 and online supplementary table 3). No time course variation in T2 value was seen in remote myocardium. Compared with remote myocardium, greater myocardial T2 value was detected in the infarct zone and remained present throughout the 3-month follow-up period (mean +12 ms, 95% CI 7 to 18, p<0.001; +14 ms, 95% CI 9 to 18, p<0.0001; +15 ms, 95% CI 6 to 24, p<0.01; +12 ms, 95% CI 5 to 19, p<0.01 and +6 ms, 95% CI 2 to 9, p<0.01 at days 1–2, 3–9, 10–16, 17–24 and 77–98, respectively).

## Discussion

For the first time, we have simultaneously investigated cellular inflammation and tissue oedema for 3 months following acute MI using USPIO-enhanced T2* and T2-mapping MRI. We have demonstrated that while tissue oedema persists for at least 3 months, cellular inflammation in the infarct zone is transient and lasts for only 2 weeks following MI. The time course of USPIO uptake following MI demonstrates a pattern of macrophage inflammation that is distinct to tissue oedema caused by the loss of capillary integrity. This suggests that while capillary integrity may take several months to resolve, cellular inflammation and specifically macrophage activity predominates in the first 2 weeks following MI.

USPIO can be used as an MRI contrast agent as it is immediately apparent in the blood pool following intravenous administration.^12^ Tissue enhancement with USPIO following inflammation has been suggested to occur as a result of either partitioning of USPIOs into the tissues because of the loss of capillary integrity alone or as a result of macrophage phagocytic activity clearing tissue-resident particles.^8^ We here provide confirmatory evidence that USPIO enhancement predominantly occurs as a result of cellular inflammation within the myocardium. We used T2 mapping to describe the loss of capillary integrity following MI. Using this comparator, we demonstrate that there is a discontinuity between the prolonged (up to 3 months) T2-defined time course of tissue oedema, and the brief (first 2 weeks) T2*-defined time-limited cellular inflammation. This observed USPIO uptake in the infarct zone for up to 2 weeks following MI is consistent with data from previous studies demonstrating that tissue-resident macrophages are active and predominate within the infarcted myocardium in the first 2 weeks after MI.^3 5^ In addition, we obtained myocardial biopsies in patients undergoing CABG surgery within 2 weeks of MI and 24 hours after USPIO administration. We demonstrated marked colocalisation of iron staining with tissue-resident macrophages in infarcted but not in non-infarcted tissue. It is not possible to resolve specific USPIOs on histology, and we cannot discount the possibility that there is some contribution from postinfarct haemosiderin staining. However, we would contend that the majority of iron staining is attributable to USPIO uptake given the selective, transient and marked R2* enhancement on MRI, and the relatively constant contribution from haemosiderin during the period of assessment. Moreover, we did not detect substantial quantities of iron that were free in the extracellular space and were not associated with areas of macrophage infiltration.

Following a single infusion in the first week after MI, USPIO enhancement is seen in all regions of the myocardium, but greatest within the infarct zone, after 24 hours. USPIO enhancement is also observed until 4–9 days in bone marrow and until 13–21 days in the liver and spleen. Therefore, the duration of USPIO enhancement is longest in the mononuclear phagocyte-rich reticuloendothelial system. It is likely that resident phagocytes within these organs ingest USPIOs from circulating blood. Efflux of myocardial macrophages containing USPIO may also contribute to the reticuloendothelial system T2* signal. Ultimately, once within macrophages, the iron oxide core is broken down by lysosomes, providing a supply of iron ions for haemoglobin synthesis.^13 14^

The observed duration of enhancement following a single infusion of USPIO in tissues helped guide the minimum time interval between repeated USPIO administrations. Complete washout of the previous dose of USPIO, allowing baseline R2* to return to normal, is required to reassess uptake of USPIO accurately, without influence from previous USPIO administration. We have shown that USPIO is no longer detectable in the myocardium at 1 week following intravenous administration, and we suggest that 1 week is the minimum interval between repeated doses of USPIO.

USPIO is detectable in the peri-infarct and remote myocardium, kidney and aortic wall at 24 hours following administration. However, the amplitude of R2* change is less than that of blood pool, suggesting this is likely to be due to a diluted effect of blood-pool USPIO and not macrophage uptake of USPIO within these tissues. Although there appears a trend to suggest that the peri-infarct zone displays an early relative increase in USPIO uptake compared with remote myocardium, this failed to reach statistical significance.

Previous studies have suggested that the remote myocardium may exhibit USPIO and macrophage accumulation.^7 8 15^ We have shown here that there is no time course variation in USPIO uptake in remote myocardium after MI, and the small R2* increase following USPIO is likely to be reflective of intravascular capillary-bed blood-pool USPIO rather than more widespread macrophage activity throughout the myocardium. This is consistent with our recent report of similar R2* increases in the myocardium of healthy volunteers which we again attribute to the blood pool signal of USPIO.^16^

Although no patients in this study displayed imaging evidence of myocardial haemorrhage, we were able to account for the presence of microscopic iron due to haemorrhage by subtracting baseline R2*. Therefore, any R2* increase detected is due to accumulation of USPIO and not iron from myocardial haemorrhage. We acknowledge that intramyocardial haemorrhage occurring between the pre and 24 hours post USPIO scans could theoretically increase R2*, but the earliest we imaged patients was at least 24 hours after MI, by which time haemorrhage is likely to have taken place.

We chose T2 mapping to assess myocardial oedema as it provides improved sensitivity and addresses some of the problems with subjective image interpretation using traditional T2-weighted techniques.^17 18^ The finding of ongoing myocardial oedema in the infarct zone at 3 months after MI may at first appear surprising. However, several groups have described the long-lasting presence of myocardial oedema, often up to 6 months after MI.^19 20^ This suggests that it may take many months for complete resolution of injury and return of normal vascular homeostasis and function.

What are the clinical applications of our technique? We have here used the ‘model’ of acute MI and demonstrated the predicted time course of tissue-resident macrophage uptake using USPIOs. We therefore suggest that this technique has utility in defining the extent of myocardial cellular inflammation that has potential utility in risk stratification. Perhaps more importantly, USPIO-enhanced MRI may prove a useful tool to assess the response to novel anti-inflammatory interventions targeted at reducing myocardial cellular inflammation to improve clinical outcomes. Such a study was carried out at our centre and has recently been published.^21^ Currently, there is no USPIO licensed as a contrast agent for routine clinical MRI, which limits its widespread use. However, the most exciting application would be to extend this technique to help diagnose, monitor disease progression and assess therapeutic intervention in myocardial diseases associated with and mediated by macrophage infiltration. Such diseases include myocarditis, cardiac transplant rejection and cardiac sarcoidosis, and a multicentre study is currently under way to assess this (EUDRACT 2013-002336-24). This would be important given the lack of simple definitive diagnostic tests in these conditions, the challenges of unreliable cardiac biopsies, and the need for rapid assessments of disease progression and therapeutic interventions.

In conclusion, we have successfully imaged, quantified and validated macrophage inflammation and myocardial oedema over 3 months in patients following MI using USPIO-enhanced T2* MRI and T2 mapping. This technique can provide a non-invasive method for the diagnosis and monitoring of tissue inflammatory macrophage activity in the heart. It also provides a potential platform on which to assess existing and novel therapeutic interventions that might modify the inflammatory process after MI and in other inflammatory processes affecting the heart.

**Key messages**

**What is already known on this subject?**

- Ultrasmall superparamagnetic particles of iron oxide (USPIO) are ingested by tissue macrophages that can be visualised using MRI to highlight areas of inflammation after myocardial infarction (MI).

**What might this study add?**

- The pattern of cellular inflammation after MI is distinct to tissue oedema. Myocardial macrophage activity is evident for 2 weeks after MI compared with the more prolonged period of myocardial oedema.

**How might this impact on clinical practice?**

- USPIO-enhanced MRI may be of value in diagnosing active MI and ultimately may provide an additional diagnostic tool or provide risk stratification and serial imaging in assessing inflammatory processes affecting the heart.

**Figure 6:**
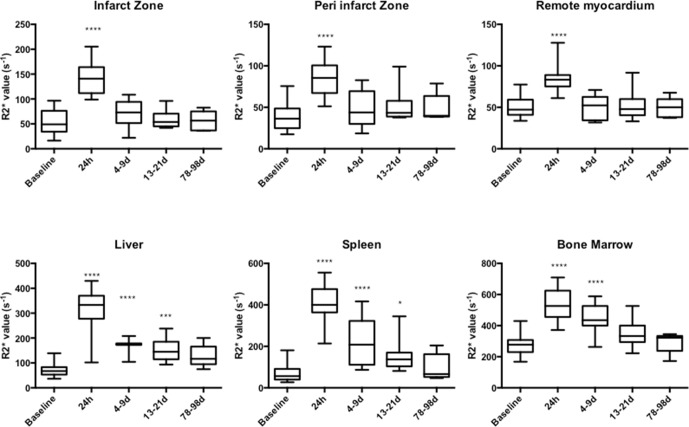
USPIO enhancement after single-dose administration in the first week following MI. Following single-dose administration, USPIO enhancement was detected in all myocardial regions at 24 hours. USPIO was detected in bone marrow until 4–9 days, and spleen and liver until 13–21 days (****p<0.0001, ***p<0.001, *p<0.05); n = 21 (baseline, 24 hours), 11 (days 4–9), 15 (days 13–21) and 5 (days 78–99). MI, myocardial infarction; USPIO, ultrasmall superparamagnetic particles of iron oxide.
