# Supplementary material for: Ferumoxytol-enhanced magnetic resonance imaging assessing inflammation after myocardial infarction
Source: Heart. 2017 Jun 22;103(19):1528–35. doi: 10.1136/heartjnl-2016-311018 (PMC5629948; doi:10.1136/heartjnl-2016-311018)
Supplement: Supplementary data [file heartjnl-2016-311018supp001.pdf]

Supplementary Material

Supplementary Methods

Magnetic Resonance Imaging Protocol

MRI was performed using a 3T scanner (Magnetom Verio, Siemens AG, Healthcare Division GmbH, Erlangen, Germany) and a Siemens 32-channel cardiac array. All images were acquired with electrocardiogram gating using expiration breath-holds. Routine steady state free precession (TrueFISP) sequences were used to acquire long- and short-axis cine images of the heart (TR 85.8 ms, TE 1.45 ms, Flip angle 50º, matrix 173x256, FoV 400 mm, slice thickness 8mm, 2mm gap). Quantitative USPIO imaging was performed again in similar slice positions using a prototype T2*-weighted multi-gradient-echo acquisition using a volumetric shim applied over the entire heart volume (TR 996 ms, TE 2.13, 4.3, 6.4, 8.6, 10.7, 12.8, 15.0, 17.1ms, Flip angle 18º, matrix 130x256, FoV 400 mm, slice thickness 6mm, 4mm gap). The T2*-weighted acquisitions included views through the liver, spleen and spine to allow quantification of USPIO accumulation within organs of the reticuloendothelial system. The same T2* protocol was used to quantify USPIO accumulation 24 h after infusion allowing calculation of T2* relaxation rates before and after administration of USPIO. oedema imaging was conducted using a Siemens T2 mapping based on a prototype T2-prepared TrueFISP acquisition acquiring identical long- and short-axis slice positions (TR 219.3 ms, TE 1.07 ms, T2 prep durations 0, 25, 50 ms, Flip angle 70º, matrix 130x192, FoV 400 mm, slice thickness 6mm, 4mm gap). T2P-TrueFisp images are

acquired at intervals of at least 3 RR intervals to allow for sufficient

magnetization recovery in between acquisitions.

Immediately after the baseline T2 and T2*-weighted scan, participants received an intravenous administration of gadolinium contrast medium (0.1 mmol/kg; Gadovist, Bayer Plc, Germany) followed by breath-held inversion recovery sequences in long axis and short axis planes to acquire late-enhancement images. Gadolinium enhanced imaging is the current gold standard for infarct detection using MRI. Optimal inversion time (TI) was determined on a slice-by-slice basis using standard late-enhancement TI-scout protocols (TR 750 ms, TE 2.61 ms, Flip angle 20º, matrix 173x256, FoV 400 mm, slice thickness 9mm, 1mm gap). The inversion-recovery late-enhancement short-axis slices were acquired using similar slice positions as the T2-edema and T2*-weighted imaging.

Histology

Myocardial tissue samples were obtained from patients undergoing coronary artery bypass graft (CABG) surgery for treatment of their coronary disease within 2 weeks of their index myocardial infarction. USPIO was administered 24 hours prior to the biopsy, and a trucut myocardial biopsy sample was taken from the infarcted area, guided by the distribution of LGE on MRI, during the procedure. The biopsy sample was fixed in formalin, embedded in paraffin, sectioned, and stained to look at architecture (hematoxylin and eosin), accumulation and distribution of USPIO (Prussian Blue) and macrophages (CD68). Patients that underwent CABG surgery were excluded from further MRI follow-up.

Supplementary Results

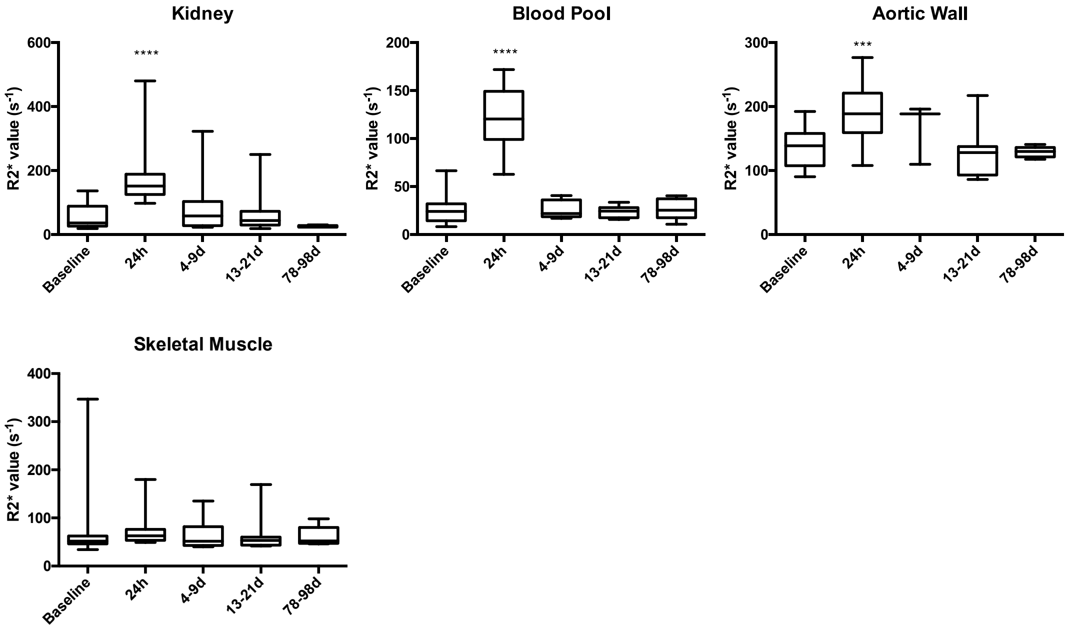

Supplementary Figure 1.

Ultrasmall superparamagnetic particles of iron oxide (USPIO) enhancement after single-dose administration in the first week following myocardial infarction in kidney, blood pool, aortic wall and skeletal muscle.

Following single-dose administration, USPIO enhancement was detected in kidney, blood pool and aortic wall at 24 hours but not thereafter (**** = p<0.0001, *** = p<0.001). There was no change in R2* in skeletal muscle following USPIO infusion (p>0.05).

Supplementary Tables

Table 1: USPIO enhancement after single-dose administration in the first week following MI.

	Baseline	24h	4-9d	13-21d	78-99d
Infarct	49 [34-76]	141 [112-164]	73 [52-94]	54 [45-70]	57 [37-75]
Peri-Infarct	36 [25-49]	85 [67-101	44 [30-69]	43 [39-58]	40 [39-64]
Remote	47 [41-59]	83 [75-89]	52 [34-63]	48 [40-60]	50 [38-60]
Liver	67 [53-83]	333 [278-370]	175 [172-178]	145 [115-185]	116 [95-166]
Spleen	56 [40-91]	400 [364-476]	208 [111-323]	137 [104-170]	66 [52-162]
Bone Marrow	278 [230-308]	526 [456-625]	435 [400-526]	333 [294-400]	323 [238-334]

Table 2a: USPIO uptake (change in R2*, s^{-1}) in myocardium after MI with repeated USPIO administration.

	2-3d	4-7d	10-16d	17-25d	78-99d
Infarct	83 [77-110]	81 [74-104]	60 [44-85]	38 [29-65]	43 [28-49]
Peri-Infarct	46 [28-63]	46 [30-64]	24 [15-46]	28 [23-45]	33 [19-48]
Remote	36 [22-41]	42 [19-50]	31 [26-39]	36 [23-40]	38 [32-49]

Table 2b: R2* (s^{-1}) 24 h following USPIO (without subtraction of baseline values).

	2-3d	4-7d	10-16d	17-25d	78-99d
Infarct	147 [109-167]	141 [116-161]	121 [81-168]	99 [82-110]	99 [82-110]
Peri-Infarct	87 [77-102]	81 [61-99]	59 [51-69]	81 [64-89]	78 [67-105]
Remote	83 [74-86]	83 [77-109]	78 [72-82]	85 [80-104]	86 [83-104]

Table 3: T2 value (ms) in the myocardium after MI.

	1-2d	3-9d	10-16d	17-24d	77-98d
Infarct	53 [48-65]	59 [56-64]	58 [54-71]	57 [50-66]	48 [45-55]
Peri-Infarct	52 [45-55]	54 [47-56]	49 [47-53]	48 [46-51]	46 [44-48]
Remote	44 [42-48]	45 [41-48]	46 [45-47]	44 [43-47]	44 [42-46}

median [IQR]
